# Twelve‐Year Clinical Course of a Band‐Shaped Remnant in the Anterior Chamber Following Trabeculotomy With the Trabectome

**DOI:** 10.1002/ccr3.72876

**Published:** 2026-06-10

**Authors:** Kazunori Takeuchi, Yuki Takagi, Ryo Asano

**Affiliations:** ^1^ Department of Ophthalmology Japan Community Healthcare Organization Chukyo Hospital Nagoya Aichi Japan

**Keywords:** band‐shaped remnant, corneal endothelial cells, glaucoma, trabectome

## Abstract

Band‐shaped remnants in the anterior chamber after trabeculotomy with the Trabectome may not be associated with long‐term clinical problems. However, it is important to be aware of the potential for a rapid decline in corneal endothelial cell density during the early postoperative period.

## Graphical abstract

Slit‐lamp and gonioscopic findings 4 months after Trabectome surgery. A band‐shaped remnant was observed in the inferonasal quadrant of the left eye, with continuity to the trabecular meshwork on gonioscopy.

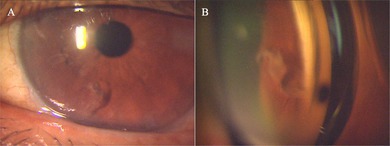

## 1 Introduction

Glaucoma is a leading cause of acquired blindness in Japan and globally. Lowering intraocular pressure (IOP) is the only proven therapeutic strategy for managing glaucoma [1, 2]. Accordingly, IOP reduction via medical therapy or surgical intervention is the standard approach [3].

In primary open‐angle glaucoma, increased resistance to aqueous humor outflow at the trabecular meshwork is the primary factor contributing to elevated IOP [4]. The Trabectome (NeoMedix, CA, USA) is a device used for performing ab interno trabeculotomy. This procedure facilitates aqueous outflow and reduces IOP by ablating a portion of the trabecular meshwork and the inner wall of Schlemm's canal with an electrocautery handpiece [5]. Trabeculotomy with the Trabectome (hereafter referred to as Trabectome surgery) is a pioneering minimally invasive glaucoma surgery. It received FDA approval in 2004, has been approved in Japan since 2010, and its efficacy has been reported by several studies [6, 7, 8, 9, 10, 11].

Trabectome surgery has been shown to reduce IOP and the need for medications for various types of glaucoma [12]. In a study of Japanese patients, IOP and medication use were reduced by 28.7% at 6 months postoperatively [6].

The known postoperative complications of Trabectome surgery include transient IOP spikes and hyphema [6, 9, 11]. Previous studies have reported no significant reduction in corneal endothelial cell density (ECD) within the first postoperative year [6, 13]. However, the long‐term impact of Trabectome surgery on ECD has not been reported. Band‐shaped remnants may occasionally appear in the anterior chamber after surgery. However, their long‐term clinical course has not been documented. We report a rare case of a band‐shaped remnant in the anterior chamber that persisted for approximately 12 years after Trabectome surgery.

## 2 Case History and Examination

A 77‐year‐old man with a history of diabetes mellitus underwent bilateral cataract extraction with intraocular lens implantation in 2008. In 2009, he underwent pars plana vitrectomy (PPV), panretinal photocoagulation (PRP), and internal limiting membrane (ILM) peeling for proliferative diabetic retinopathy with macular edema in the left eye. His condition remained stable after these interventions. He was treated with oral circulation‐improving agents and topical antiglaucoma medications for diabetic macular edema in the left eye and secondary glaucoma in both eyes.

In March 2012, rhegmatogenous retinal detachment was diagnosed in the right eye. He underwent PPV with PRP, ILM peeling, and intraocular air tamponade. The postoperative course was uneventful. However, elevated IOP persisted in the left eye and had been noted prior to the surgery. Adequate IOP reduction was not achieved despite treatment with three topical antiglaucoma agents (tafluprost, carteolol hydrochloride, and brinzolamide) and oral acetazolamide (250 mg, two tablets daily). The elevated IOP in the left eye was attributed to secondary open‐angle glaucoma following vitrectomy, and Trabectome surgery was performed as planned.

## 3 Methods

A 1.7‐mm temporal corneal incision was made in the left eye, followed by a 120‐degree nasal trabecular meshwork incision. This incision range is consistent with the commonly used approach for Trabectome surgery, in which approximately 90–120 degrees of nasal trabecular meshwork is typically ablated [7, 12]. Intraoperative bleeding was observed at the incision site. Hemostasis was achieved as much as possible using standard irrigation and aspiration techniques. A band‐shaped remnant tissue continuous with the trabecular meshwork was observed in the inferonasal quadrant of the anterior chamber after the incision. The remnant was not removed. The procedure was completed after confirming that the IOP had decreased to below 20 mmHg. The medication score for IOP control was calculated as follows: one point was assigned for each topical single‐agent antiglaucoma medication, two points for each fixed‐combination topical medication, and one point for oral acetazolamide. In addition, corneal ECD was measured preoperatively and postoperatively to evaluate longitudinal changes using a CEM‐530 specular microscope (NIDEK, Aichi, Japan) at the approximate center of the cornea.

## 4 Conclusions and Results

**FIGURE 1:**
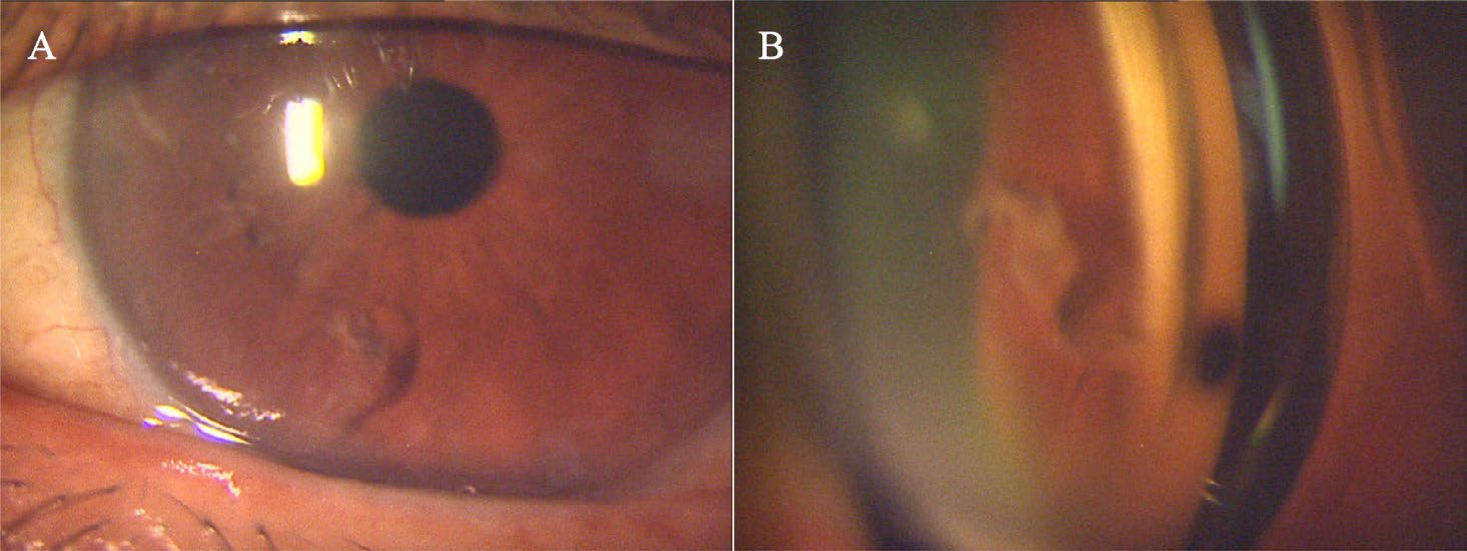
The slit‐lamp findings for the anterior segment and angle at 4 months postoperatively are shown. (A) A band‐shaped remnant with an approximate length of 4 mm observed in the inferonasal quadrant of the left eye after Trabectome surgery. (B) Gonioscopic examination revealed continuity between the remnant and the trabecular meshwork in the inferonasal angle quadrant.

**FIGURE 2:**
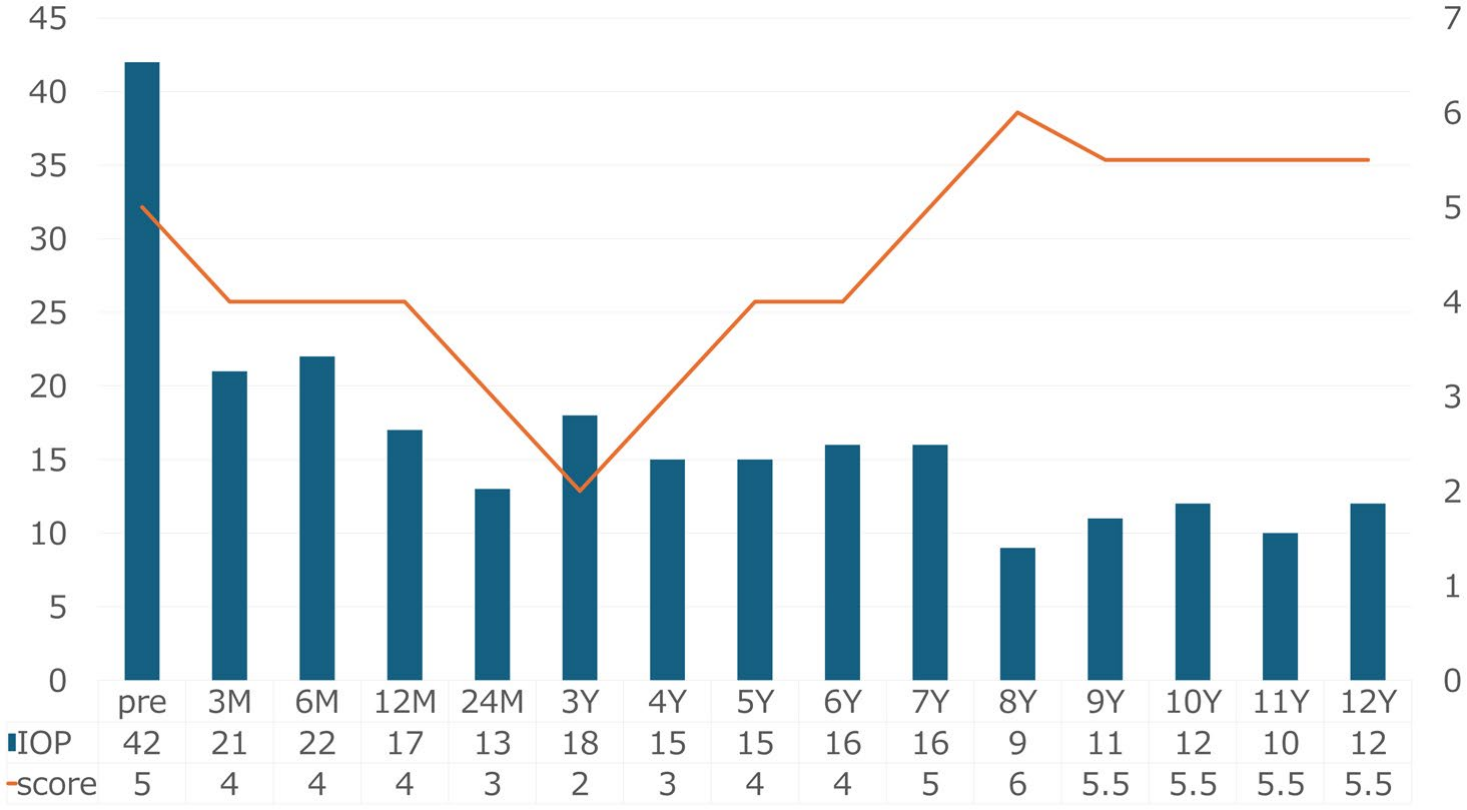
Changes in IOP and the medication score over the 12 years postoperatively. The medication score decreased from 5 preoperatively to 2 at 3 years postoperatively. However, it had increased to 5.5 at 12 years. The IOP reduction rate over the 12‐year postoperative period was 71.4%.

A band‐shaped remnant with a length of approximately 4 mm was observed in the inferonasal anterior chamber postoperatively. Gonioscopy confirmed its continuity with the trabecular meshwork (Figure 1). The IOP decreased to the low teens immediately after surgery. The medication score increased over the long term, but the IOP reduction was substantial throughout the follow‐up period (Figure 2). No additional glaucoma surgeries were required.

**FIGURE 3:**
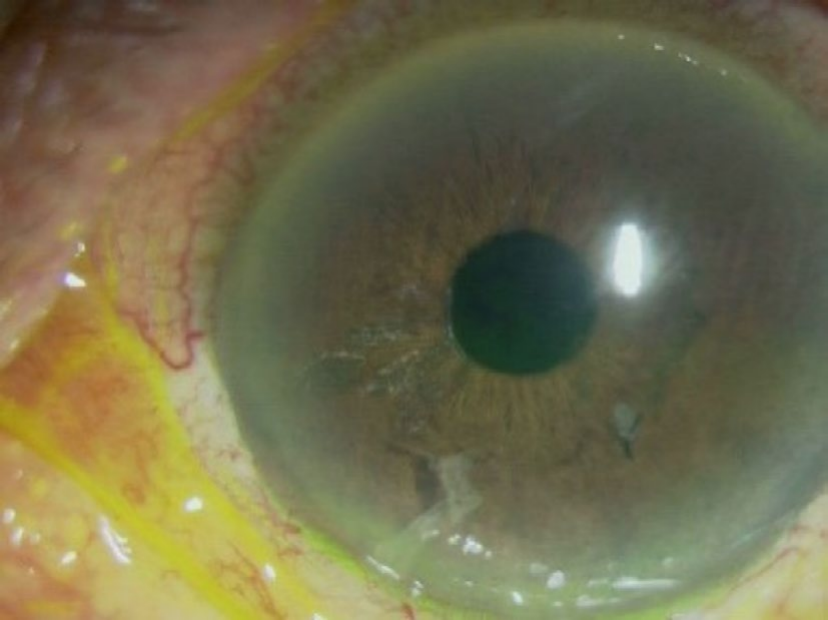
Slit‐lamp findings 12 years postoperatively: Anterior segment. No morphological changes in the band‐shaped remnant or intraocular inflammation were observed during the 12‐year follow‐up. Adhesion between the tip of the remnant and the iris was observed 12 years postoperatively.

The corneal ECD decreased from 2370 cells/mm^2^ preoperatively to 1821 cells/mm^2^ at 6 months postoperatively. However, it did not change significantly thereafter (1856 cells/mm^2^ at 12 years postoperatively). No morphological changes in the band‐shaped remnant or signs of anterior chamber inflammation were observed during follow‐up. Adhesion between the tip of the remnant and the iris was observed at 12 years postoperatively (Figure 3).

## 5 Discussion

We observed a band‐shaped remnant in the anterior chamber following Trabectome surgery and monitored its progression for 12 years. The remnant remained unchanged throughout the follow‐up, with no signs of regression, dissolution, or inflammation. Its origin is presumed to be ablated trabecular meshwork tissue; however, this remains hypothetical because histopathological confirmation was not obtained. Although transient hyphema and IOP spikes are known postoperative findings after Trabectome surgery, detailed reports describing the long‐term clinical course of a persistent band‐shaped remnant in the anterior chamber are lacking. Residual lens cortex after cataract surgery can induce inflammation and often requires removal. However, ablated trabecular tissue, as in this case, may not provoke inflammation and may be managed conservatively with careful observation in selected cases.

The medication score increased during follow‐up. However, the IOP remained below 20 mmHg, representing a reduction of more than 20% from baseline. Previous studies have reported IOP reductions of approximately 28.7% to 38.0% [6, 11]. The IOP reduction rates in our case were 50% at 1 year and 71.4% at 12 years postoperatively. This case involved secondary open‐angle glaucoma following vitrectomy, and postoperative outcomes may differ from those in primary open‐angle glaucoma because of differences in the underlying pathophysiology. Although Trabectome surgery has been reported to be effective in both primary and secondary open‐angle glaucoma, 12 reports focusing specifically on secondary glaucoma after vitrectomy are limited. Therefore, the favorable IOP course in this case should be interpreted cautiously. Nevertheless, the long‐term IOP control observed in this case suggests that the remnant did not substantially impede aqueous outflow through the trabecular meshwork.

The reduction in the corneal ECD in this patient was approximately 23.5% at 6 months postoperatively, which is more severe than those reported in previous studies. Physiological ECD loss is estimated at approximately 0.6% per year [14]. However, follow‐up at 1 year after Trabectome surgery revealed a reduction in central ECD of only approximately 0.8% per year with no significant decline [13]. Therefore, the reduction rate in this case was relatively high. This early postoperative decline was likely multifactorial and cannot be attributed solely to the presence of the remnant. Conversely, the preoperative ECD was not decreased, and no further ECD decline was observed from 6 months postoperatively to 12 years despite persistence of the remnant. These findings suggest that factors occurring in the perioperative or early postoperative period may have had a greater influence on the initial ECD decline than preexisting ocular conditions alone.

Several factors may account for the initial postoperative decline. First, surgical trauma associated with the Trabectome procedure itself may have contributed. Endothelial damage to the peripheral cornea near the incision or trabecular ablation site may have contributed to the reduction in central ECD at 6 months. Second, previous vitreoretinal surgery and diabetic retinal disease may have increased the vulnerability of the corneal endothelium, although these factors alone do not fully explain the abrupt postoperative decline because the preoperative ECD was preserved. Third, measurement variability cannot be excluded in a retrospective single‐case observation. Mechanical trauma from the remnant is another possibility. The remnant may have been more mobile during the early postoperative phase and may have intermittently contacted the corneal endothelium, contributing to ECD loss. The adhesion of the remnant to the iris may have reduced its mobility over the long term and helped minimize further endothelial injury. Importantly, despite the remnant remaining in the anterior chamber without removal, ECD remained stable for more than 10 years after the early postoperative decline.

These observations highlight the importance of regular monitoring of the ECD in cases with remnants after Trabectome surgery. Removal of the remnant may be considered to prevent progressive endothelial injury secondary to mechanical trauma if significant ECD loss is observed early postoperatively or persists. Conversely, the remnant may pose minimal long‐term risk to the corneal endothelium if ECD remains stable after the early postoperative period. Observation without intervention may be reasonable for selected cases, provided that careful long‐term monitoring is maintained.

This report describes only a single case; therefore, the findings should be interpreted with caution. Future studies involving multiple cases are needed to further investigate the long‐term effects of such remnants on IOP, ECD changes, and their morphological evolution. We hypothesize that the remnant tissue in this case originated from the ablated trabecular meshwork. However, its histological characteristics have not been determined. Therefore, excision of the remnant and histopathological analysis may provide valuable insights for similar cases when removal is clinically indicated.

We have reported a case involving the persistence of a band‐shaped remnant in the anterior chamber after Trabectome surgery that was monitored for 12 years. The remnant showed no significant morphological changes or signs of inflammation during the long‐term follow‐up, suggesting that careful observation may be a reasonable management option in selected cases. However, regular monitoring of ECD is recommended because a rapid decline in ECD during the early postoperative period may occur and may be multifactorial.

## Author Contributions

**Kazunori Takeuchi:** conceptualization, investigation, visualization, writing – original draft. **Yuki Takagi:** conceptualization, data curation, investigation, supervision, visualization, writing – review and editing. **Ryo Asano:** conceptualization, supervision.

## Funding

The authors have nothing to report.

## Consent

The patient provided written informed consent for the publication of her examination and imaging findings for educational, research, and quality improvement purposes.

## Conflicts of Interest

The authors declare no conflicts of interest.

## Data Availability

The data supporting the findings of this study are available from the corresponding author upon reasonable request. Due to patient privacy considerations, the data are not publicly available. Some longitudinal data are partially unavailable due to the retrospective nature of follow‐up.

## References

[1] R. N. Weinreb , T. Aung , and F. A. Medeiros , “The Pathophysiology and Treatment of Glaucoma: A Review,” Journal of the American Medical Association 311, no. 18 (2014): 1901–1911, 10.1001/jama.2014.3192. 24825645 PMC4523637

[2] Y. C. Tham , X. Li , T. Y. Wong , H. A. Quigley , T. Aung , and C. Y. Cheng , “Global Prevalence of Glaucoma and Projections of Glaucoma Burden Through 2040: A Systematic Review and Meta‐Analysis,” Ophthalmology 121, no. 11 (2014): 2081–2090, 10.1016/j.ophtha.2014.05.013. 24974815

[3] M. A. Kass , D. K. Heuer , E. J. Higginbotham , et al., “The Ocular Hypertension Treatment Study:A Randomized Trial Determines That Topical Ocular Hypotensive Medica Tion Delays or Prevents the Onset of Primary Open‐ Angle Glaucoma,” Archives of Ophthalmology 120, no. 6 (2002): 701–713; discussion 829, 10.1001/archopht.120.6.701. 12049574

[4] R. Rosenquist , D. Epstein , S. Melamed , M. Johnson , and W. M. Grant , “Outflow Resistance of Enucleated Human Eyes at Two Different Perfusion Pressures and Different Extents of Trabeculotomy,” Current Eye Research 8, no. 12 (1989): 1233–1240, 10.3109/02713688909013902. 2627793

[5] B. A. Francis , R. F. See , N. A. Rao , D. S. Minckler , and G. Baerveldt , “Ab Interno Trabeculectomy: Development of a Novel Device (Trabectome) and Surgery for Open‐Angle Glaucoma,” Journal of Glaucoma 15, no. 1 (2006): 68–73, 10.1097/01.ijg.0000196653.77836.af. 16378021

[6] M. Masahiro , W. Mitsunori , and K. Ichikawa , “Evaluation of Trabectome in Open‐Angle Glaucoma,” Journal of Glaucoma 22 (2013): 205–208. 23429629 10.1097/IJG.0b013e3182311b92

[7] K. Kitamura , Y. Fukuda , Y. Hasebe , M. Matsubara , and K. Kashiwagi , “Mid‐Term Results of Ab Interno Trabeculectomy Among Japanese Glaucoma Patients,” Journal of Clinical Medicine 12, no. 6 (2023): 2332, 10.3390/jcm12062332. 36983331 PMC10055689

[8] Y. Kono , M. Kasahara , K. Hirasawa , et al., “Long‐Term Clinical Results of Trabectome Surgery in Patients With Open‐Angle Glaucoma,” Graefe's Archive for Clinical and Experimental Ophthalmology 258, no. 11 (2020): 2467–2476, 10.1007/s00417-020-04897-0. 32857189

[9] W. Constance , L. Elisabeth , H. Sarah , et al., “Five‐Year Clinical Outcomes of Inferior Quadrant Trabectome Surgery for Open Angle Glaucoma,” Journal of Glaucoma 32 (2023): 480–488. 36930581 10.1097/IJG.0000000000002164

[10] T. Naoki , “Atsushi: The Outcomes of Trabectome Surgery in Patients With Low, Middle, and High Preoperative Intraocular Pressure,” Clinical Ophthalmology 14 (2020): 4099–4108. 33273806 10.2147/OPTH.S285883 PMC7708680

[11] R. A. Widder , M. Hild , T. S. Dietlein , et al., “Trabectome, Trabecular Aspiration and Phacoemulsification in a Triple Procedure for Treating Exfoliation Glaucoma: A Long‐Term Follow‐Up,” European Journal of Ophthalmology 31, no. 5 (2021): 2432–2438, 10.1177/1120672120956505. 32914642

[12] J. F. Jordan , T. Wecker , C. van Oterendorp , et al., “Trabectome Surgery for Primary and Secondary Open Angle Glaucomas,” Graefe's Archive for Clinical and Experimental Ophthalmology 251, no. 12 (2013): 2753–2760, 10.1007/s00417-013-2500-7. PMC3889259 24158374

[13] M. Kasahara , N. Shoji , and K. Matsumura , “The Influence of Trabectome Surgery on Corneal Endothelial Cells,” Journal of Glaucoma 28, no. 2 (2019): 150–153, 10.1097/IJG.0000000000001128. 30394978

[14] W. M. Bourne , L. R. Nelson , and D. O. Hodge , “Central Corneal Endothelial Cell Changes Over a Ten‐Year Period,” Investigative Ophthalmology & Visual Science 38, no. 3 (1997): 779–782. 9071233

